# Moderate muscle cooling induced by single and intermittent/prolonged cold-water immersions differently affects muscle contractile function in young males

**DOI:** 10.3389/fphys.2023.1172817

**Published:** 2023-03-21

**Authors:** Viktorija Treigyte, Nerijus Eimantas, Tomas Venckunas, Marius Brazaitis, Thomas Chaillou

**Affiliations:** ^1^ Sports Science and Innovation Institute, Lithuanian Sports University, Kaunas, Lithuania; ^2^ School of Health Sciences, Örebro University, Örebro, Sweden

**Keywords:** cold exposure, cold-water immersion, muscle contractility, muscle fatigue, cold habituation, muscle force, temperature

## Abstract

**Background:** We investigated the impact of moderate muscle cooling induced by single and intermittent/prolonged cold-water immersions (CWI) on muscle force and contractility in unfatigued state and during the development of fatigue resulting from electrically induced contractions.

**Methods:** Twelve young males participated in this study consisting of two phases [single phase (SP) followed by intermittent/prolonged phase (IPP)], with both phases including two conditions (i.e., four trials in total) performed randomly: control passive sitting (CON) and cold-water immersions (10°C). SP-CWI included one 45 min-bath (from 15 to 60 min). IPP-CWI included three baths (45 min-bath from 15 to 60 min, and 15 min-baths from 165 to 180 min and from 255 to 270 min), with participants sitting at room temperature the rest of the time until 300 min. Blood pressure and intramuscular (Tmu) temperature were assessed, and neuromuscular testing was performed at baseline and 60 min after baseline during SP, and at baseline, 60, 90, 150 and 300 min after baseline during IPP. A fatiguing protocol (100 electrical stimulations) was performed after the last neuromuscular testing of each trial.

**Results:** In unfatigued state, SP-CWI and IPP-CWI reduced electrically induced torque at 100 Hz (P100) but not at 20 Hz (P20), and increased P20/P100 ratio. The changes from baseline for P100 and P20/P100 ratio were lower in IPP-CWI than SP-CWI. Both cold-water immersion conditions slowed down muscle contraction and relaxation, and reduced maximal isokinetic contraction torque, but the changes from baseline were lower after IPP-CWI than SP-CWI. cold-water immersions did not impair maximal voluntary isometric contraction. During the fatiguing protocol, torque fatigue index and the changes in muscle contractile properties were larger after IPP-CWI than SP-CWI, but were in the same range as after CON conditions. The differences of muscle contractile function between SP-CWI and IPP-CWI were accompanied by a lower reduction of superficial Tmu and a smaller increase in systolic blood pressure after IPP-CWI than SP-CWI.

**Conclusion:** IPP-CWI induces a less pronounced fast-to-slow contractile transition compared to SP-CWI, and this may result from the reduced vasoconstriction response and enhanced blood perfusion of the superficial muscle vessels, which could ultimately limit the reduction of superficial Tmu.

## 1 Introduction

Application of cooling is commonly used in public health to reduce heat strain and improve thermal comfort in hot environment (Lan et al., 2018; Tokizawa et al., 2020), as well as to improve wellbeing and to potentially reduce inflammation in patients (Tipton et al., 2017). Cooling is also applied before and during exercise in hot conditions combined or not with high humidity to attenuate exertional heat strain and optimize physical performance (Coelho et al., 2021; Morito et al., 2022). Moreover, post-exercise cooling such as cold-water immersion (CWI) has become very popular in athletes based on the assumption that it could enhance physical recovery and reduce muscle soreness (Yeargin et al., 2006; Brophy-Williams et al., 2011; Machado et al., 2017). Cold exposure also occurs in numerous sports (e.g., winter sports, open-water swimming, etc.) and occupational activities (e.g., military expedition, emergency rescue, etc.). Severe and/or prolonged cooling can have deleterious effects, such as hypothermia, cold-injuries, and impaired physical performance (Cahill et al., 2011; Fudge et al., 2015).

Exposure to CWI induces acute physiological adjustments, including increased whole-body metabolic heat production (Castellani et al., 1998), reduced muscle metabolic activity (Ihsan et al., 2013), reduced femoral artery blood flow and increased cutaneous vasoconstriction (Mawhinney et al., 2017), reduced muscle and core temperatures (Brazaitis et al., 2014b), and decreased nerve conduction velocity (Algafly and George, 2007). These adjustments are influenced by the duration of exposure, water temperature, and the type of immersion (e.g., single, or intermittent) (Brazaitis et al., 2011; Brazaitis et al., 2014b; Castellani and Young, 2016).

Cooling could also influence skeletal muscle function (Bennett, 1984; Oksa, 2002; Drinkwater, 2008). Numerous human studies have demonstrated that CWI-induced severe muscle cooling (i.e., reduced temperature of 10°C–20°C in the deep portion of the muscle) impairs electrically evoked and maximal voluntary isometric contraction (MVIC) forces (Davies et al., 1982; Giesbrecht et al., 1995; de Ruiter et al., 1999). Severe muscle cooling also elicits a shift towards a slower muscle contractile profile (Davies et al., 1982; de Ruiter et al., 1999), while it could limit force decline during repeated contractions induced by electrical stimulation (Davies et al., 1982). In contrast, moderate muscle cooling, that can be defined as a reduced temperature up to 5°C in the deep portion of the muscle, does not (Petrofsky and Phillips, 1986) or only slightly (Asmussen et al., 1976; Bergh and Ekblom, 1979; Brazaitis et al., 2011) reduces MVIC force. Some evidence indicates that it reduces force during dynamic voluntary contractions, at least of the knee extensors (Bergh and Ekblom, 1979), and force response to 50 Hz electrical stimulations (Brazaitis et al., 2011). However, it may not affect the drop in force (i.e., fatigue resistance) and half relaxation time (HRT) during repeated electrical stimulations induced contractions (Brazaitis et al., 2011). To date, the impact of moderate cooling on muscle force production (isometric and dynamic contractions, and electrically evoked contractions at low and high frequencies) and muscle contractile properties (including rate of force development, rate of force relaxation, contraction time and HRT) in unfatigued state is not well documented. In addition, the effect of moderate cooling on muscle force and contractile properties during the development of fatigue resulting from electrical stimulations requires further investigation.

Human studies evaluating the impact of local cooling on muscle force and contractile properties generally include one single CWI where the immersion time is either not documented [but adjusted so that skeletal muscle reaches a certain temperature (Asmussen et al., 1976; Bergh and Ekblom, 1979)] or limited to a duration <45 min (Davies et al., 1982; de Ruiter et al., 1999; Brazaitis et al., 2011; Brazaitis et al., 2012). To investigate physiological changes in response to prolonged muscle cooling (i.e., several hours), intermittent water immersions can be used to limit cold sensation (Castellani et al., 1998). Single exposure to CWI markedly increases heat production related to shivering and non-shivering thermogenesis and limits heat loss through cutaneous vasoconstriction, while intermittent CWI immersions possibly result in thermoregulatory adjustments and habituation, including reduced shivering and/or cutaneous vasoconstriction response (Castellani and Young, 2016). This latter physiological response may increase the supply of warm blood from the core to the skin and superficial layers of the muscles, while maintaining a reduced temperature in the deep portion of the muscle. In that case, it could be hypothesized that intermittent and prolonged CWI would lead (in comparison to shorter single CWI) to a faster muscle contractile profile in unfatigued state and during the development of fatigue resulting from electrically induced contractions. Intermittent and prolonged CWI may also attenuate the force reduction during dynamic voluntary contractions in unfatigued state due to the faster contractile properties of the muscle (and thus its ability to attain peak force more rapidly), while MVIC would be unaffected. Furthermore, voluntary activation in unfatigued state would most likely not be affected by short and prolonged CWI, as evidenced in recent studies (Brazaitis et al., 2014b; Spillane and Bampouras, 2021). In this study, our aim was to investigate the impact of moderate muscle cooling induced by single and intermittent/prolonged CWI on muscle force production and muscle contractility in unfatigued state and during the development of fatigue resulting from electrically induced contractions in humans.

## 2 Material and methods

### 2.1 Participants

A randomized cross-over design was used in this study, in which twelve recreationally active men participated. The inclusion criteria used were: aged between 18 and 45 years, not participating in any other experiments, being healthy, physically active (at least 2–3 times per week) and without medication, and having a body mass index (BMI) < 30 kg.m-2. The exclusion criteria were: asthma, neurological pathology, cardiovascular disease, or conditions that could be worsened by exposure to cold, and suffering from any kind of disease or having physical limitations that would compromise the ability to perform the neuromuscular testing. The age, height, body mass, percentage body fat and BMI assessed at baseline at the beginning of the study were 27.2 ± 6.6 years, 186.7 ± 7.6 cm, 86.5 ± 12.1 kg, 16.9% ± 3.1% and 24.9 ± 2.2 kg/m2, respectively. All experiments were performed at the Lithuanian Sports University (Kaunas, Lithuania). The study protocol was approved by Kaunas Regional Biomedical Research Ethics committee (no. P1-BE-2–14/2022) and was in agreement with the latest revision of the Declaration of Helsinki. The participants were informed of the experimental procedures and gave their written informed consent prior to participation.

### 2.2 Experimental design

**FIGURE 1:**
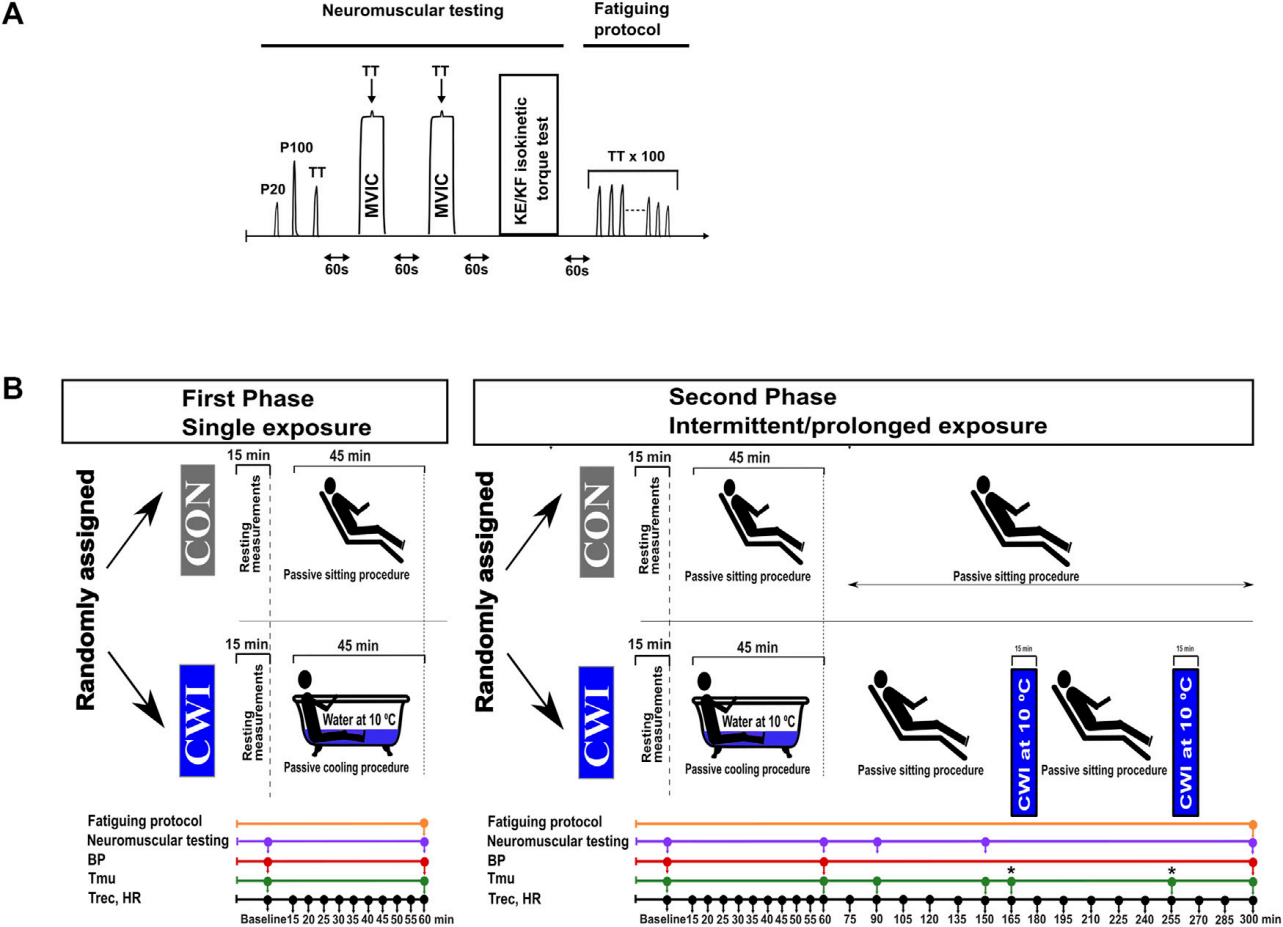
Neuromuscular testing and fatiguing protocol **(A)**, and experimental design **(B)**. P20, electrical stimulation at 20 Hz; P100, electrical stimulation at 100 Hz; TT, 250-ms test train stimulation at 100 Hz; MVIC, maximal voluntary isometric contraction; KE, knee extension; KF, knee flexion; BP, blood pressure; Tmu, intramuscular temperature; Trec, rectal temperature; HR, heart rate; CON, control condition; CWI, cold-water immersion condition. Fatiguing protocol consisting of 100 trains of electrical stimulation of the knee extensors (100 x 250-ms TT) was only performed after the last neuromuscular testing (i.e., second neuromuscular testing during the single phase and 5th neuromuscular testing during the intermittent/prolonged phase). *: at the time points 165 and 255 min, Tmu was only measured in the CWI condition.

The study included two phases: the phase “single exposure”, called single phase (SP), followed by the phase “intermittent/prolonged exposure”, called intermittent/prolonged phase (IPP), with both phases consisting of two experimental conditions performed in a random order: control sitting (CON) and cold-water immersion (CWI). Each experimental trial (four in total) was separated by at least 1 week. A summary of the experimental design is presented in Figure 1. The week before the first experiment, the participants were familiarized with the protocols, equipment, the neuromuscular testing, and fatiguing protocol (see description below). Air temperature in the laboratory was controlled and maintained at 23.0°C ± 0.5 °C, and relative humidity was 35% ± 3%.

The participants arrived at the laboratory and started the experiments in the morning (9.00 a.m.) after overnight fasting. Body mass, body composition, heart rate (HR), blood pressure (BP), rectal temperature (Trec) and intramuscular temperature (Tmu) were assessed at baseline (after sitting for 15 min). Then, they performed the first neuromuscular testing, which was followed by either 45 min control passive sitting in the laboratory (CON condition) or by 45 min sitting in a cold-water bath (CWI condition, immersion up to the waist in an acrylic bathtub, water temperature of 10.0°C ± 0.2°C). Ice slush was added into the bath to maintain the required water temperature. HR and Trec were recorded every 5 min during this period. Then, HR, BP, Trec and Tmu were measured, and the second neuromuscular testing (endpoint during the single phase and at 60 min during the intermittent/prolonged phase) followed. During the single phase, the second neuromuscular testing was directly followed by a fatiguing protocol consisting of 100 trains of electrical stimulation of the knee extensors (Table 1; see below for details). During IPP-CWI, two additional bouts of cold-water immersion (15 min each, immersion up to the waist, 10.0°C ± 0.2°C) were added between 165 and 180 min, and between 255 and 270 min. During this experimental trial, participants were sitting at room temperature the rest of the time: between 60 and 165 min (duration of 105 min), between 180 and 255 min (duration of 75 min), and between 270 and 300 min (duration of 30 min). During IPP-CON, participants were sitting at room temperature for 300 min. During the intermittent/prolonged phase, HR and Trec were recorded every 15 min between 60 min and the end of the protocol (i.e., 300 min), and BP was measured at the end. Tmu was measured at 90 min, 150 min and at the end of CON condition (300 min), and two additional Tmu measurements were added after 165 min (before the second bath) and 255 min (before the third bath) in the IPP-CWI condition to monitor possible changes in Tmu due to passive sitting between baths. The number of Tmu measurements was limited to limit discomfort and tissue damage. Additional neuromuscular testing was performed at 90 min, 150 min, and at the end of the intermittent/prolonged phase. After the last neuromuscular testing, a fatiguing protocol consisting of 100 trains of electrical stimulations of the knee extensors was directly performed.

**TABLE 1:** Changes in the physiological measurements after the single and intermittent/prolonged phases.

	SP-CON	IPP-CON	P	*d*	SP-CWI	IPP-CWI	P	*d*
Δ HR (bpm)	−2.50 ± 8.42	−0.67 ± 5.1	0.60	−0.15	−4.67 ± 6.07	−6.33 ± 7.05	0.52	0.19
Δ Diastolic BP (mmHG)	−0.17 ± 7.31	0.83 ± 7.35	0.74	−0.10	2.17 ± 9.92	2.92 ± 5.82	0.80	−0.07
Δ Systolic BP (mmHG)	−0.08 ± 8.56	2.67 ± 11.42	0.54	−0.18	7.08 ± 10.34	0.17 ± 5.46	0.04	0.65
Δ Trec (°C)	−0.01 ± 0.17	−0.18 ± 0.34	0.12	0.48	−0.01 ± 0.17	−0.78 ± 0.37	<0.001	1.80
Δ Tmu (°C) at 3 cm	−0.05 ± 0.42	−0.66 ± 0.63	0.02	0.88	−3.78 ± 1.89	−4.29 ± 1.17	0.34	0.30
Δ Tmu (°C) at 2 cm	−0.38 ± 0.65	−0.79 ± 1.29	0.29	0.34	−6.09 ± 1.72	−4.57 ± 1.25	0.02	−0.81
Δ Tmu (°C) at 1 cm	−0.50 ± 0.73	−0.65 ± 0.81	0.62	0.15	−9.73 ± 2.66	−4.83 ± 1.30	<0.001	−1.96

*d*, Cohen’s *d*, HR, heart rate; BP, blood pressure; Trec, rectal temperature; Tmu, intramuscular temperature. SP, single phase; IPP, intermittent/prolonged phase.

Data are shown as mean ± SD. Δ change was calculated as: end value—baseline value.

N = 12 for all parameters except for Tmu (N = 11).

During the intermittent/prolonged phase, a strawberry breakfast cereal bar (87 kcal; Fitness, South Africa) was provided at 120 min and 225 min, while the participants remained fasted during the single phase. Participants were told to take similar meals on the day before experiments. During the experiments, participants did not drink water, but were allowed to rinse their mouth with cool water. Participants wore only swimming shorts during the baths, and quickly wiped off at the end of each bath before starting the neuromuscular testing. Between the baths (only for intermittent/prolonged cooling) and in the CON conditions, they wore shorts and a tee-shirt.

### 2.3 Physiological measurements

#### 2.3.1 Anthropometric measurements

Body mass and percentage body fat were assessed with a body composition analyzer (Tanita, TBF-300, IL, United States). The height of the participants was measured with a height gauge, and BMI was calculated.

#### 2.3.2 Blood pressure and heart rate measurements

Diastolic and systolic BP were measured on the left arm (one measurement each time) with an automatic BP monitor (Gentle+, Microlife, FL, United States). HR was recorded with a HR monitor (S-625X, Polar, Kempele, Finland). The time points of measurements are presented in the section “experimental design” and in Figure 1.

#### 2.3.3 Body temperature measurements

Trec and Tmu were similarly assessed as in some studies from our research group (Brazaitis et al., 2011; Brazaitis et al., 2014b) and the time points are presented in the section “experimental design” and in Figure 1. More precisely, Trec was measured using a thermocouple (Rectal Probe; Ellab, Hvidovre, Denmark; accuracy, ±0.01°C) inserted to a depth of 12 cm past the anal sphincter. Tmu was measured using a needle microprobe (Intramuscular Probe MKA, thermometer model DM-852, Ellab) inserted into the vastus lateralis muscle of the right leg at mid-thigh and slightly lateral to the femur at three different depths (1, 2 and 3 cm beneath the skin surface).

### 2.4 Rationale of the CWI protocols

**FIGURE 2:**
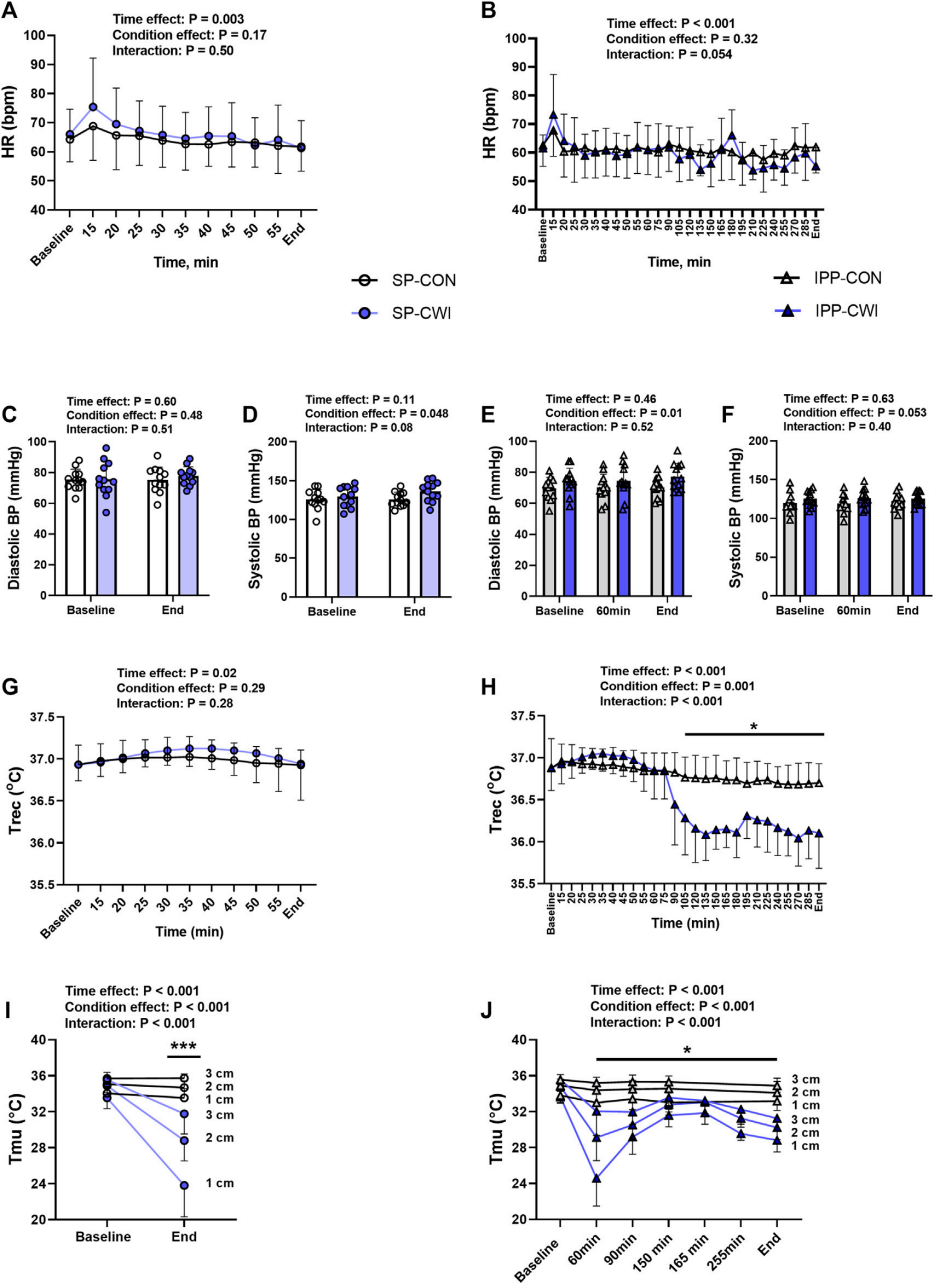
Physiological measurements during the single and intermittent/prolonged phases. Heart rate (HR) **(A,B)**, diastolic **(C,E)** and systolic **(D,F)** blood pressures (BP), rectal temperature (Trec) **(G,H)** and intramuscular temperature (Tmu) at three depths (1, 2, and 3 cm) **(I,J)** during the single and intermittent/prolonged phases, respectively. Data are shown as mean ± SD and individual values are presented in panels C–F. SP, single phase; IPP, intermittent/prolonged phase. *, *p* < 0.05; ***, *p* < 0.001: Significant differences CON vs. CWI. In I and J, significant differences are between CON and CWI are at the three depths. In J, Tmu was not assessed in CON at the time points 165 and 255 min. N = 12 for all parameters except for Tmu (N = 11) **(I,J)**.

In this study, we developed our CWI protocols with the objective of inducing a moderate reduction of Tmu (<5°C) in the deep portion of the vastus lateralis muscle. We defined deep Tmu at a depth of 3 cm in the vastus lateralis muscle, which is commonly used in studies investigating force in the knee extensors in response to CWI (Asmussen et al., 1976; Bergh and Ekblom, 1979; Beelen and Sargeant, 1991; Brazaitis et al., 2011; Brazaitis et al., 2012). Previous studies and data from our laboratory have shown that after a relatively short exposure to CWI (10 min), deep Tmu continues to decrease for at least 30–40 min post-immersion (Mawhinney et al., 2013; Mawhinney et al., 2017; Eimonte et al., 2021). To maintain a relatively stable deep Tmu after the first immersion and during the second neuromuscular testing (and during the fatiguing protocol of SP), the first bath was set for 45 min. Based on pilot experiments and as illustrated in Figure 2J, we found that deep Tmu remained relatively stable for approximately 100 min following the first 45 min-CWI. For this reason, two additional 15 min-CWI were included during IPP: one at 165-min (i.e., 105 min after the first 45 min-bath and directly after the fourth neuromuscular testing) and one at 255 min. The duration of these two baths was only of 15 min to limit participants’ discomfort and to maintain Trec higher than 35.5°C, a set point previously used in studies from our group (Brazaitis et al., 2014a; Brazaitis et al., 2014b; Brazaitis et al., 2015).

### 2.5 Neuromuscular testing, fatiguing protocol and data analysis

Neuromuscular testing, consisting of electrically evoked torque of the knee extensors, maximal voluntary isometric contraction (MVIC) of the knee extensors, and maximal isokinetic concentric contraction of the knee extensors (KE-isoK) and knee flexors (KF-isoK) is illustrated in Figure 1A. Involuntary contractions and voluntary contractions were previously performed in studies from our research group (Skurvydas et al., 2011; Brazaitis et al., 2014b; Eimantas et al., 2022). More precisely, the participants sat upright in an isokinetic dynamometer (System 4; Biodex Medical Systems, Shirley, NY, United States) calibrated according to the manufacturer’s recommendations, with a correction for gravity performed using the Biodex Advantage program (Version 4. X). Shank, trunk and shoulders were stabilized with belts. The dynamometer was set with the knee joint positioned at an angle of 90° (180° = full extension) during MVIC and electrical stimulations, and at a joint angle between 85° and 176° during KE-isoK and between 176° and 85° during KF-isoK.

Electrical stimulations were applied using three carbonized rubber surface electrodes (MARP Electronic), lubricated with electrode gel (ECG-EEG Gel, medigel, Modi’in, Israel). Two electrodes (12 × 8 cm) were positioned vertically and transversely across the width of the proximal portion of quadriceps muscle, and the third electrode (12 × 8 cm) covered the distal portion of the quadriceps muscle above the patella. Caution was made to keep a similar position of the electrodes during the four experimental trials. An electrical stimulator (Digitimer DS7A, Digitimer, Hertfordshire, United Kingdom) was connected to the electrodes and delivered 0.5-ms square wave pulses at a constant current set at 100 mA and constant voltage limit set at 200 V. This selected current ensures full contraction and activation of the muscle (Eimantas et al., 2022).

Neuromuscular testing began with three electrical stimulations separated with 3 s of rest: 1-s stimulation at 20 Hz (P20), 1-s stimulation at 100 Hz (P100) and 250-ms test train stimulation at 100 Hz (TT). Peak torques were determined during these three electrical stimulations, and P20/P100 was calculated to estimate the contractile profile. The contractile properties in the unfatigued state were determined during TT by calculating contraction time/peak torque, half-relaxation time (HRT), peak rate of torque development (RTD) and peak rate of torque relaxation (RTR). Contraction time was defined as the time taken to reach peak torque. Contraction time progressively decreased in our fatiguing protocol (see the description below) as a result of the progressive reduction of torque production and thus the fact that it takes less time to reach a lower torque (data not shown). Consequently, we chose to normalize contraction time to peak torque in both protocols (i.e., unfatigued state and fatiguing protocol). This ratio represents the average time required to increase the torque by 1 Nm. HRT was calculated as the time taken for the torque to decline from the peak value to 50% of the peak value. RTD and RTR were calculated by using Excel software and raw data exported from Biodex system (100Hz sampling rate) and were defined as the peak slope of torque per 10 ms (Δtorque/Δ10 ms) (Eimantas et al., 2022).

Following these three electrical stimulations, two MVIC of the knee extensor muscle were performed and separated by 1-min rest period. The participants were verbally encouraged to exert and maintain maximal effort for ∼5 s, and a 250-ms test train stimulation at 100 Hz was superimposed on voluntary contraction 3–4 s into the MVIC. Central activation ratio (CAR), a measure of voluntary activation level, was calculated as (MVIC torque/total peak torque generated with the superimposed 250-ms test train stimulation) x 100. The highest MVIC torque was selected for analysis. One minute after the second MVIC, three continuous repetitions of KE-isoK (90°/s) and KF-isoK (90°/s) were performed with 1 min rest between KE and KF contractions. The highest torque during these maximal isokinetic contractions was selected for analysis.

Directly after the last neuromuscular testing (i.e., second during the single phase and 5th during the intermittent/prolonged phase), a fatiguing protocol consisting of 100 trains of electrical stimulation of the knee extensor muscle [250-ms test train stimulation at 100 Hz (TT) interspaced with 1s break] was included. TT torque and the contractile properties (contraction time/peak torque, HRT, RTD and RTR) were determined during the fatiguing protocol and are presented as the average of the first three contractions, 4th to 20th contraction, 21st to 40th contraction, 41st to 60th contraction, 61st to 80th contraction, and 81st to 100th contraction. The torque fatigue index and the changes in contractile properties during fatiguing protocol were determined from the first three and last three contractions (Table 2).

**TABLE 2:** Torque fatigue index and changes in contractile properties during the fatiguing protocol.

	SP-CON	IPP-CON	P	*d*	SP-CWI	IPP-CWI	P	*d*
Torque fatigue index (%)	54.4 ± 13.4	56.4 ± 12.4	0.16	−0.44	49.8 ± 13.0	55.5 ± 11.7	0.01	−0.86
Contraction time/peak torque (% change)	126.4 ± 81.2	129.5 ± 90.7	0.79	−0.08	92.0 ± 76.3	121.8 ± 77.2	0.02	−0.82
HRT (% change)	125.1 ± 81.0	121.4 ± 60.2	0.78	0.08	66.7 ± 60.1	108.1 ± 73.2	0.01	−0.88
RTD (% change)	−58.8 ± 17.3	−57.8 ± 15.8	0.77	−0.10	−53.5 ± 16.5	−58.2 ± 15.2	0.08	0.62
RTR (% change)	−75.3 ± 11.0	−74.9 ± 9.4	0.86	−0.06	−60.5 ± 12.9	−66.2 ± 20.8	0.19	0.45

*d*, Cohen’s *d*, HRT, half-relaxation time; RTD, peak rate of torque development; RTR, peak rate of torque relaxation; SP, single phase; IPP, intermittent/prolonged phase.

Data are shown as mean ± SD., Torque fatigue index was calculated as: [(average of the first 3 contractions—average of the last 3 contractions)/average of the first 3 contractions] x 100.% change was calculated as: [(average of the last 3 contractions—average of the first 3 contractions)/average of the first 3 contractions] x 100.

N = 12 for all parameters except for RTD, and RTR (N = 10).

### 2.6 Statistical analysis

**FIGURE 3:**
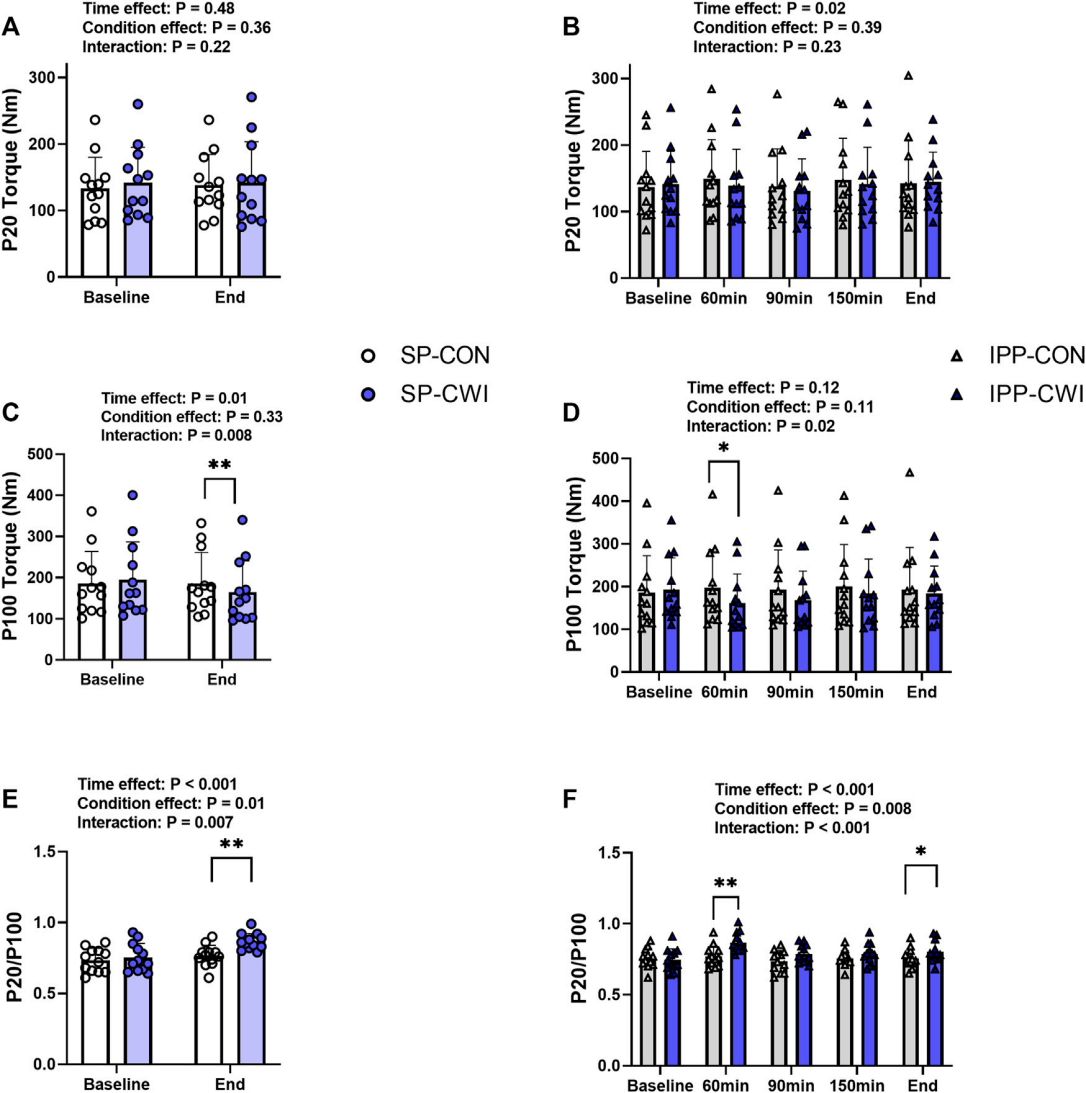
Electrically induced isometric torques in the unfatigued state. Peak torques at 20 Hz (P20) **(A,B)** and 100 Hz (P100) **(C,D)**, and P20/P100 **(E,F)** during the single and intermittent/prolonged phases, respectively. Data are shown as mean ± SD and all panels include individual values. SP, single phase; IPP, intermittent/prolonged phase. *, *p* < 0.05; **, *p* < 0.01: Significant differences CON vs. CWI. N = 12.

**FIGURE 4:**
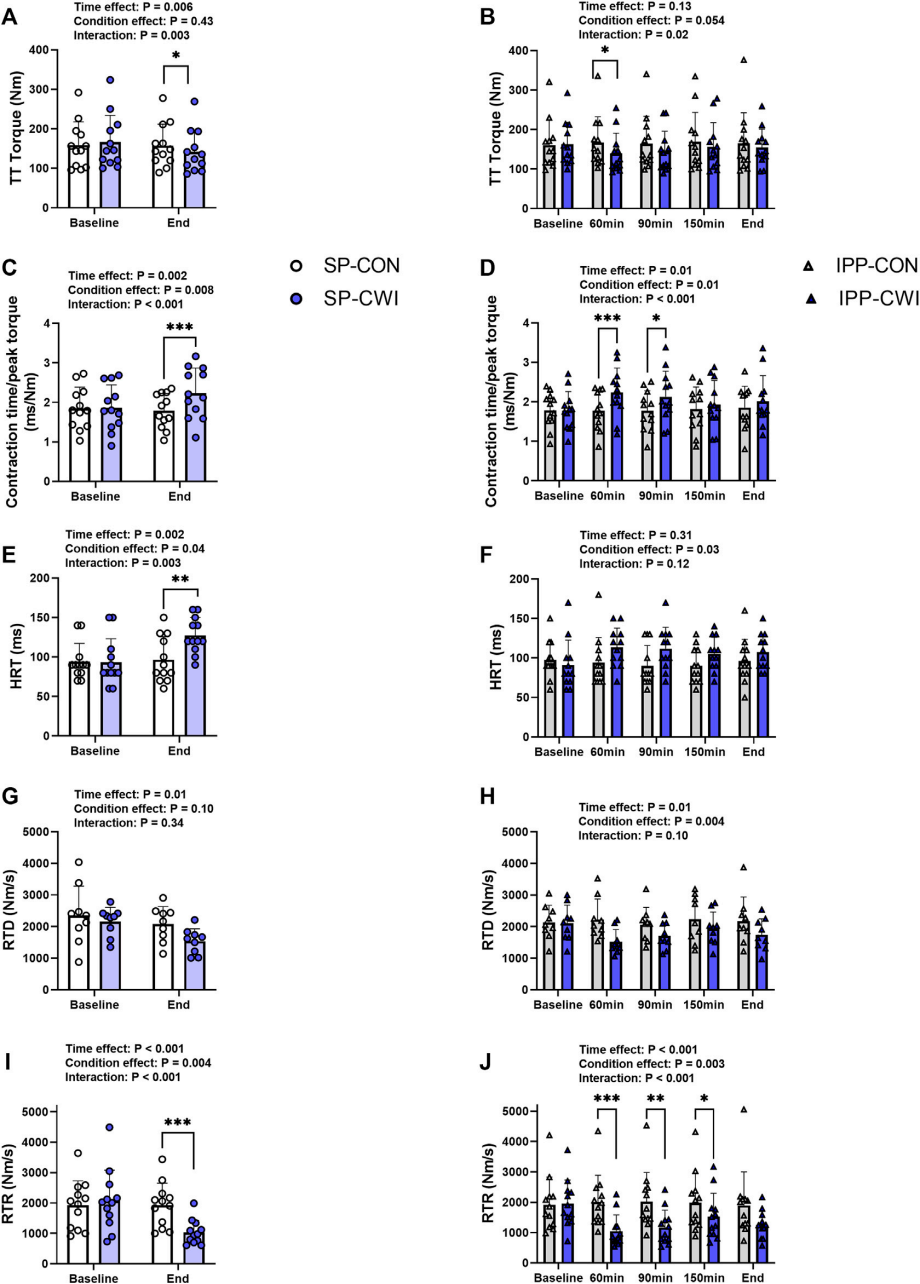
Peak torques and contractile properties derived from a 250-ms test train stimulation at 100 Hz (TT) in the unfatigued state. TT torques **(A,B)**, contraction time/peak torque **(C,D)**, half-relaxation time (HRT) **(E,F)**, peak rate of torque development (RTD) **(G,H)** and peak rate of torque relaxation (RTR) **(I,J)** during the single and intermittent/prolonged phases, respectively. Data are shown as mean ± SD and all panels include individual values. SP, single phase; IPP, intermittent/prolonged phase. *, *p* < 0.05; **, *p* < 0.01; ***, *p* < 0.001: Significant differences CON vs. CWI. N = 12 for all parameters except for RTD (N = 9) **(G,H)**.

**FIGURE 5:**
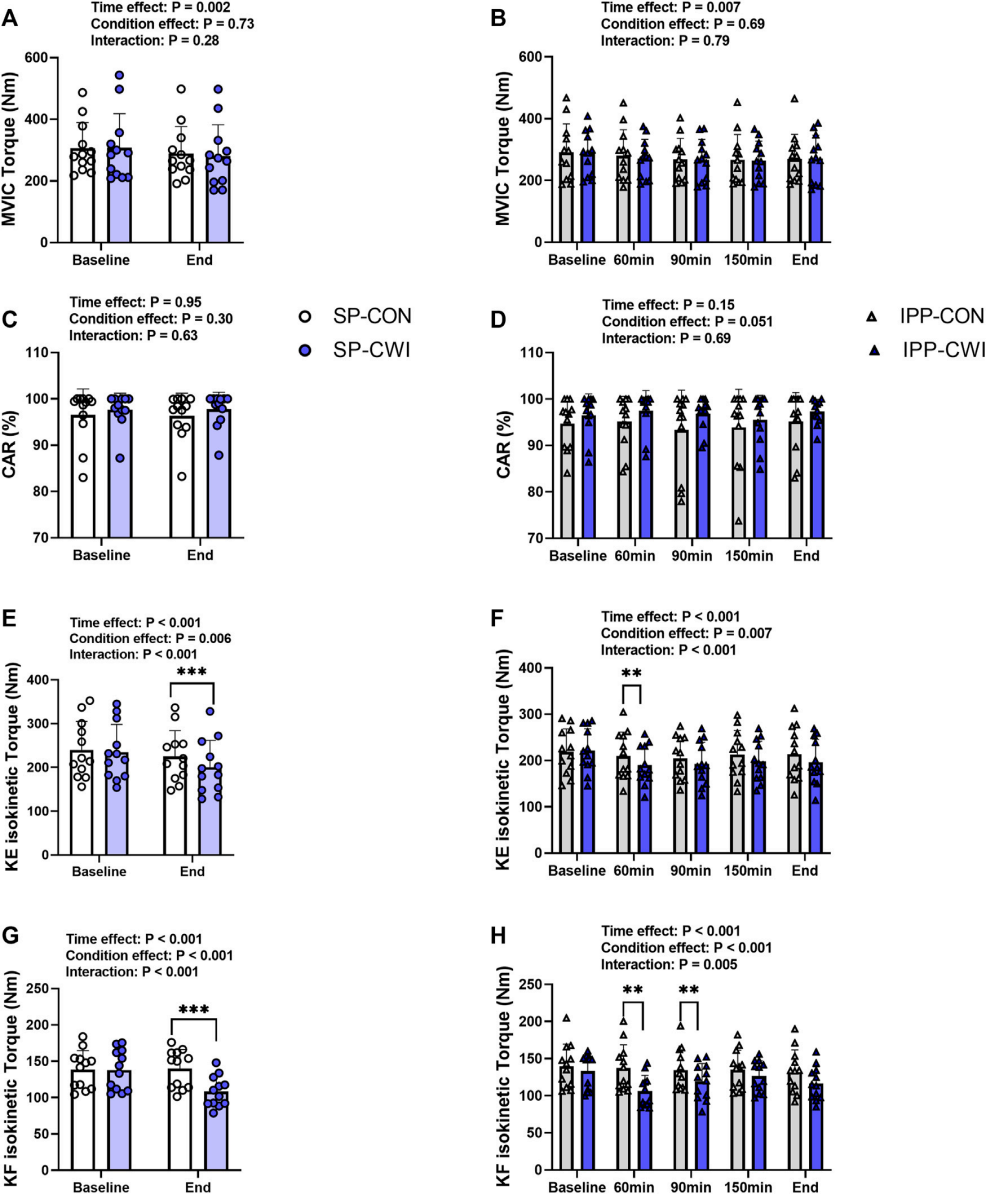
Maximal voluntary contraction torques and central activation ratio (CAR) in the unfatigued state. Maximal voluntary isometric contraction (MVIC) torques **(A,B)**, CAR **(C,D)**, knee extension (KE) maximal isokinetic torques **(E,F)**, and knee flexion (KF) maximal isokinetic torques **(G,H)** during the single and intermittent/prolonged phases, respectively. Data are shown as mean ± SD and all panels include individual values. SP, single phase; IPP, intermittent/prolonged phase. **, *p* < 0.01; ***, *p* < 0.001: Significant differences CON vs. CWI. N = 12.

**FIGURE 6:**
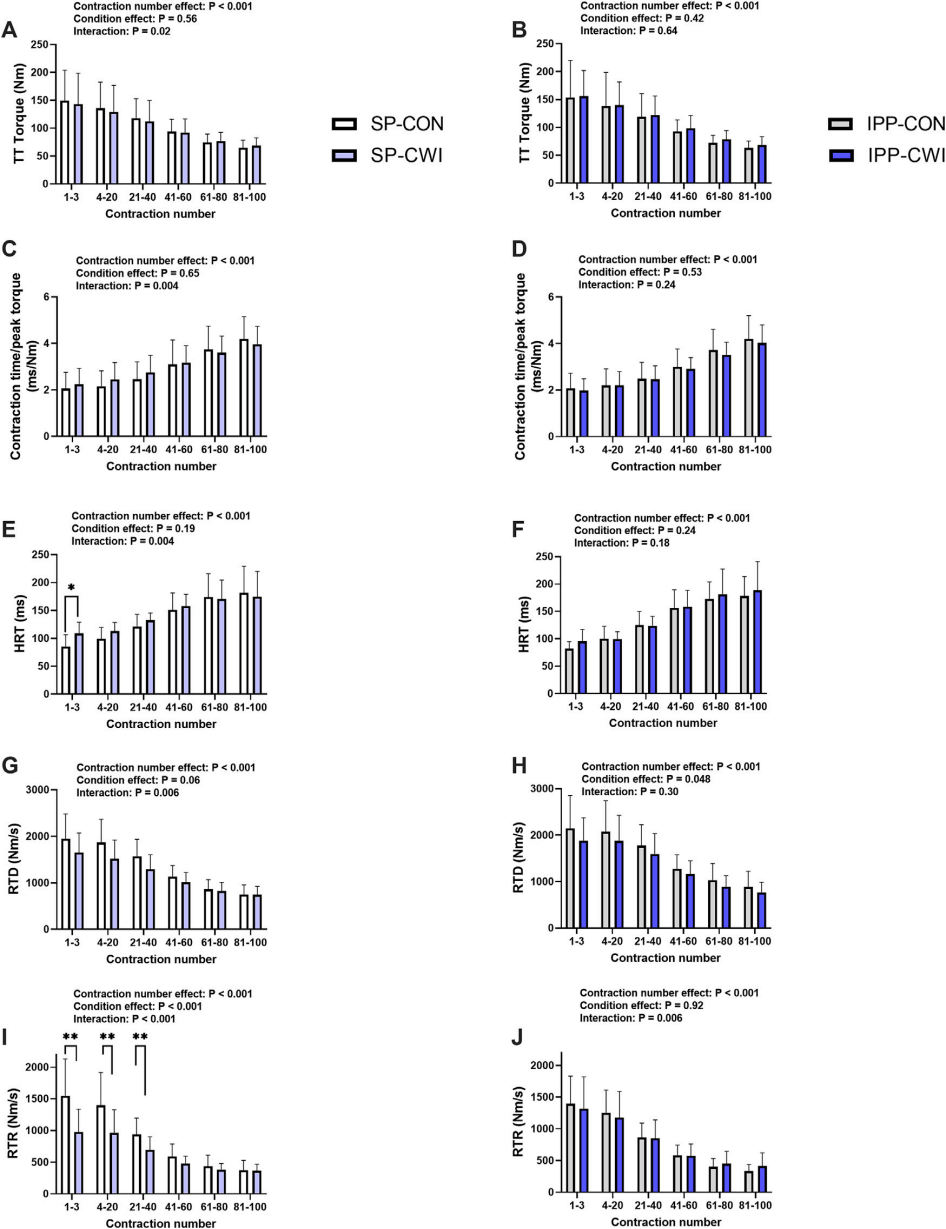
Torques and contractile properties during the fatiguing protocol. Torques during 250-ms test train stimulations at 100 Hz (TT torques) **(A,B)**, contraction time/peak torque **(C,D)**, half-relaxation time (HRT) **(E,F)**, peak rate of torque development (RTD) **(G,H)**, and peak rate of torque relaxation (RTR) **(I,J)** during the single and intermittent/prolonged phases, respectively. Data are shown as mean ± SD. SP, single phase; IPP, intermittent/prolonged phase. *, *p* < 0.05; **, *p* < 0.01: Significant differences CON vs. CWI. N = 12 for all parameters except for RTD (N = 10) **(G,H)** and RTR (N = 10) **(I,J)**.

Data are presented as mean ± standard deviation (SD) and data presented in figures also include individual values (except in some panels of Figures 2, 6). Statistical analyses were performed using GraphPad Prism (Graphpad Prism 9.0.2, San Diego, CA, United States) and SPSS Statistics (version 28; for analysis of effect size only). Data were tested for normality using the Shapiro–Wilk test before conducting parametric statistical analyses, and all data were found to be normally distributed. For the physiological parameters (HR, BP, Trec and Tmu; Figure 2), and torques and contractile properties in unfatigued state (Figure 3; Figure 4; Figure 5), two-way repeated-measures analyses of variance (ANOVA) were performed to assess the effects of condition (CON vs. CWI), time and the condition × time interaction during both the single and intermittent/prolonged phases. When an interaction was observed, Sidak’s multiple comparisons test was used to compare the two conditions (CON vs. CWI). For the physiological parameters (Table 1) and torques and contractile properties in unfatigued state (Table 3; Table 4), the Δ change (i.e., end value—baseline value) and % change (i.e [(end value—baseline value)/baseline value] x 100) between SP-CON and IPP-CON, and between SP-CWI and IPP-CWI were assessed using paired t-tests. For TT torque and the contractile properties during the fatiguing protocol (Figure 6), two-way repeated-measures ANOVA were performed to assess the effects of condition (CON vs. CWI), contraction number (1–3, 4–20, 21–40, 41–60, 61–80, 81–100) and the condition x contraction number interaction during both phases. When an interaction was observed, Sidak’s multiple comparisons test was used to compare the two conditions (CON vs. CWI). In Supplementary Table S1, two-way repeated-measures ANOVA were also performed to assess the effects of the phase (single vs. intermittent/prolonged), contraction number (1–3, 4–20, 21–40, 41–60, 61–80, 81–100) and the phase x contraction number interaction. When an interaction was observed, Sidak’s multiple comparisons test was used to compare the two phases for both CON (SP-CON vs. IPP-CON) and CWI (SP-CWI vs. IPP CWI) conditions. All two-way repeated-measures ANOVA included Geisser-Greenhouse corrections to correct for violation of the sphericity assumption. To compare the torque fatigue index and the changes in contractile properties during fatiguing protocol between SP-CON and IPP-CON, and between SP-CWI and IPP-CWI (Table 2), paired t-tests were used. Three participants were excluded from the analysis of RTD in the unfatigued state, and two participants were excluded from the analysis of RTD and RTR during the fatiguing protocol due to technical issues. One participant was also excluded from the analysis of Tmu due to incorrect measurements. The α-level of significance was set at *p* < 0.05. Partial eta squared (η_p_^2^) was determined to estimate the effect size for the two-way repeated-measures ANOVA. Cohen’s *d* was calculated to interpret the magnitude of the mean difference between two conditions, and effect sizes were classified as small (│*d*│ from 0.2 to 0.5), moderate (│*d*│ from 0.5 to 0.8) and large (│*d*│ above 0.8).

**TABLE 3:** Changes in torques and contractile properties in the unfatigued state.

	SP-CON	IPP-CON	P	*d*	SP-CWI	IPP-CWI	P	*d*
Δ P20 torque (Nm)	5.13 ± 15.87	5.70 ± 20.50	0.95	−0.02	0.03 ± 11.92	3.46 ± 17.17	0.54	−0.18
Δ P100 torque (Nm)	−0.02 ± 27.99	6.84 ± 27.09	0.65	−0.13	−30.29 ± 19.71	−9.34 ± 25.87	0.02	−0.79
P20/P100 (% change)	4.24 ± 7.01	0.28 ± 4.81	0.14	0.46	16.00 ± 10.96	7.95 ± 5.55	0.041	0.67
Δ TT torque (Nm)	−1.01 ± 14.39	4.68 ± 18.63	0.49	−0.20	−24.12 ± 18.50	−8.37 ± 20.80	0.047	−0.64
Δ Contraction time/peak torque (ms/Nm)	−0.06 ± 0.23	0.07 ± 0.15	0.16	−0.43	0.37 ± 0.19	0.24 ± 0.28	0.17	0.42
Δ HRT (ms)	2.50 ± 20.50	−1.67 ± 29.18	0.66	0.13	34.17 ± 21.51	16.67 ± 18.26	0.01	0.89
Δ RTD (Nm/s)	−270.0 ± 945.3	−36.7 ± 449.2	0.36	−0.32	−625.6 ± 158.1	−361.1 ± 225.9	0.007	−1.19
Δ RTR (Nm/s)	2.5 ± 287.3	−25.8 ± 385.9	0.88	0.05	−1041.7 ± 724.8	−691.7 ± 408.4	0.03	−0.74

*d*, Cohen’s *d*, P20, electrically induced isometric torque at 20 Hz; P100, electrically induced isometric torque at 100 Hz; TT, 250-ms test train stimulation at 100 Hz; HRT, half-relaxation time; RTD, peak rate of torque development; RTR, peak rate of torque relaxation; SP, single phase; IPP, intermittent/prolonged phase.

Contractile properties (i.e., contraction time/peak torque, HRT, RTD, and RTR) are derived from the TT, stimulation. Data are shown as mean ± SD. Δ change was calculated as: end value—baseline value. % change was calculated as: [(end value—baseline value)/baseline value] x 100.

N = 12 for all parameters except for RTD (N = 9).

**TABLE 4:** Changes in maximal voluntary contraction torques and central activation ratio in the unfatigued state.

	SP-CON	IPP-CON	P	*d*	SP-CWI	IPP-CWI	P	*d*
Δ MVIC torque (Nm)	−16.96 ± 15.84	−21.38 ± 32.93	0.66	0.13	−26.58 ± 28.64	−16.55 ± 16.90	0.26	−0.35
CAR (% change)	−0.12 ± 2.14	0.49 ± 4.16	0.67	−0.13	−0.14 ± 1.17	0.94 ± 4.15	0.58	−0.16
Δ KE isokinetic torque (Nm)	−15.03 ± 15.23	−5.55 ± 15.20	0.14	−0.45	−33.85 ± 13.13	−24.69 ± 9.99	0.01	−0.90
Δ KF isokinetic torque (Nm)	1.37 ± 11.88	−7.10 ± 10.37	0.08	0.56	−29.76 ± 10.69	−16.58 ± 15.60	0.008	−0.93

*d*, Cohen’s *d*, MVIC, maximal voluntary isometric contraction; CAR, central activation ratio; KE, knee extension; KF, knee flexion; SP, single phase; IPP, intermittent/prolonged phase.

Data are shown as mean ± SD. Δ change was calculated as: end value—baseline value. % change was calculated as: [(end value—baseline value)/baseline value] x 100.

N = 12.

## 3 Results

### 3.1 Physiological measurements

HR slightly decreased during the single and intermittent/prolonged phases (time effect during SP: *p* = 0.003, η_p_^2^ = 0.31; time effect during IPP: *p* < 0.001, η_p_^2^ = 0.36; Figures 2A,B) but was not affected by cooling. Diastolic BP was higher in CWI than CON during the intermittent/prolonged phase (condition effect: *p* = 0.01, η_p_^2^ = 0.44; Figure 2E), but not during the single phase (Figure 2C). In addition, a significant condition effect was found for systolic BP during the single phase (*p* = 0.048, η_p_^2^ = 0.31; Figure 2D), while it almost reached the level of significance during the intermittent/prolonged phase (*p* = 0.053, η_p_^2^ = 0.30; Figure 2F). Trec remained roughly constant in both the CON and CWI conditions during the single phase and during the first 60 min of the intermittent/prolonged phase (Figures 2G,H). During the latter phase, Trec decreased in the CWI condition from 105 min and remained lower compared with the CON condition until the end (*p* < 0.05, d: 1.21 to −2.74). Tmu (at the three depths) were similarly reduced in CWI compared with CON at the end of the single phase and at 60 min during the intermittent/prolonged phase (*p* < 0.05, d: −1.18 to −2.89; Figures 2I,J). Tmu (at the three depths) remained lower in CWI than in CON until the end of the intermittent/prolonged phase (*p* < 0.05, d: 1.18 to −2.73). However, Tmu at 1 and 2 cm depth varied in CWI from 60 min to the end of the intermittent/prolonged phase. The Δ changes from baseline (i.e., end values—baseline values) were not different between the single and intermittent/prolonged phases in the CON condition for any of these physiological parameters, except for Tmu at 3 cm depth (*p* = 0.02, *d* = 0.88) (Table 1). The Δ changes for systolic BP, Trec, and Tmu (1 and 2 cm depth) were lower in IPP-CWI than in SP-CWI (*p* < 0.05, *d* reported in Table 1), while the Δ changes between these two conditions were not significantly different for HR, diastolic BP, and Tmu at 3 cm depth.

### 3.2 Electrically induced torques and contractile properties in the unfatigued state

Representative 20 Hz (P20) and 100 Hz (P100) torques are illustrated in Supplementary Figure S1A and results are presented in Figure 3. P20 torques remained constant during the single phase and slightly increased during the intermittent/prolonged phase (time effect, *p* = 0.02, η_p_^2^ = 0.25), but they were not affected by the cooling condition (Figures 3A,B). P100 torques were lower in CWI than in CON at the end of the single phase (*p* < 0.01, *d* = −1.13; Figure 3C) and only at 60 min during the intermittent/prolonged phase (*p* < 0.05, *d* = −0.95; Figure 3D). As a result, P20/P100 ratio increased in CWI compared with CON at the end of the single phase (*p* < 0.01, *d* = 1.28) and at 60 min during the intermittent/prolonged phase (*p* < 0.01, *d* = 1.57; Figures 3E,F). P20/P100 ratio was not different between CON and CWI at both 90 and 150 min during the intermittent/prolonged phase, while it became higher again in CWI than in CON at the end of this phase (*p* < 0.05, *d* = 0.98). The Δ changes from baseline (for P20 and P100 torques) and the % changes from baseline (for P20/P100 ratio) were not different between SP-CON and IPP-CON (Table 3). The Δ changes for P20 torques were not different between SP-CWI and IPP-CWI. However, the Δ changes for P100 torques and the % changes for P20/P100 ratio were larger in SP-CWI than in IPP-CWI (P100 torque: *p* = 0.02, *d* = −0.79; P20/P100 ratio: *p* = 0.041, *d* = 0.67).

A 250-ms test train stimulation (TT) was performed after P100 to assess the contractile properties in the unfatigued state. Representative TT torques are illustrated in Supplementary Figure S1 and results of TT and contractile properties are presented in Figure 4. Similar to P100 torques, TT torques were reduced in CWI compared with CON at the end of the single phase (*p* < 0.05, *d* = −0.96; Figure 4A) and only at 60 min during the intermittent/prolonged phase (*p* < 0.05, *d* = −1.11; Figure 4B). Contraction time/peak torque ratio was higher in CWI than in CON at the end of the single phase (*p* < 0.001, *d* = 1.51; Figure 4C) and after 60 min (*p* < 0.001, *d* = 1.63) and 90 min (*p* < 0.05, *d* = 1.05) during the intermittent/prolonged phase (Figure 4D). HRT was also greater in CWI compared with CON at the end of the single phase (*p* < 0.01, *d* = 1.25; Figure 4E). A significant condition effect was found for HRT during the intermittent/prolonged phase (*p* = 0.03, η_p_^2^ = 0.36; Figure 4F) without any significant condition × time interaction (*p* = 0.12, η_p_^2^ = 0.17). A significant condition effect was also found for RTD during the intermittent/prolonged phase (*p* = 0.004, η_p_^2^ = 0.67; Figure 4H) while only a tendency was observed during the single phase (*p* = 0.10, η_p_^2^ = 0.31; Figure 4G), without any significant condition × time interaction in both phases. RTR highly decreased in CWI compared with CON at the end of the single phase (*p* < 0.001, *d* = −1.59; Figure 4I) and at 60, 90, and 150min during the intermittent/prolonged phase (*p* < 0.05, d: 0.91 to −1.60; Figure 4J). The Δ changes from baseline were not different between SP-CON and IPP-CON conditions for TT torque, contraction time/peak torque ratio, HRT, RTD, and RTR (Table 3). Except for contraction time/peak torque ratio, the Δ changes for the other parameters of contractile properties were lower in IPP-CWI than in SP-CWI conditions (*p* < 0.05, *d* reported in Table 3).

### 3.3 Maximal voluntary contraction torques in the unfatigued state

Voluntary torque production was assessed from maximal voluntary isometric contraction (MVIC) and maximal isokinetic contraction of the knee extensors (KE-isoK) and knee flexors (KF-isoK). MVIC torques slightly decreased during the single (time effect, *p* = 0.001, η_p_^2^ = 0.62; Figure 5A) and intermittent/prolonged phases (time effect, *p* = 0.006, η_p_^2^ = 0.36; Figure 5B) but were not affected by cooling. CAR remained constant in both the CON and CWI conditions during the intermittent/prolonged phase (Figures 5C,D). KE-isoK torques were lower in CWI than in CON at the end of the single phase (*p* < 0.001, *d* = −1.50; Figure 5E) and only at 60min during the intermittent/prolonged phase (*p* < 0.01, *d* = −1.17; Figure 5F). KF-isoK torques decreased in CWI compared with CON at the end of the single phase (*p* < 0.001, *d* = −2.12; Figure 5G) and a similar result was found at 60 min (*p* < 0.01, *d* = −1.44) and 90 min during the intermittent/prolonged phase (*p* < 0.01, *d* = −1.21; Figure 5H). The Δ changes from baseline (for MVIC, KE-isoK torques, and KF-isoK torques) and the % changes from baseline (for CAR) were not different between SP-CON and IPP-CON conditions (Table 4). The Δ changes for MVIC torques and the % changes for CAR were not different between SP-CWI and IPP-CWI conditions, while the Δ changes for KE-isoK and KF-isoK torques were larger in SP-CWI than in IPP-CWI (KE-isoK torque: *p* = 0.01, *d* = −0.90; KF-isoK torque: *p* = 0.008, *d* = −0.93) (Table 4).

### 3.4 Electrically induced torques and contractile properties during fatiguing protocol

A fatiguing protocol, consisting of 100 electrical stimulations of the knee extensor muscle [250-ms test train stimulation at 100 Hz (TT) interspaced with 1-s break], was performed at the end of the single (i.e., at 60 min) and intermittent/prolonged (i.e., 300 min) phases. TT torques and the contractile properties were determined, and results are presented in Figure 6, Supplementary Table S1 and in Table 2. Representative torques are illustrated in Supplementary Figure S1B. Decreases in TT torques, RTD and RTR, and increases in contraction time/peak torque ratio and HRT were found over the fatiguing protocol in CON and CWI conditions in both phases, as illustrated in Figure 6 (contraction number effect, *p* < 0.001, η_p_^2^: 0.72–0.84) and Supplementary Table S1, and in Table 2. Significant condition x contraction number were found for all these parameters during the single phase (*p* < 0.02, η_p_^2^: 0.35–0.76), but not during the intermittent/prolonged phase (except for RTR, *p* = 0.006, η_p_^2^ = 0.40) (Figure 6). In addition, two-way repeated-measures ANOVA revealed significant phase x contraction number interactions for all these parameters in the CWI condition (*p* < 0.02), but not in the CON condition (Supplementary Table S1). During the single phase, *post hoc* analyses detected higher HRT during the first 3 contractions in CWI compared with CON (*p* < 0.05, *d* = 1.08; Figure 6E), and lower RTR during the first 40 contractions in CWI compared with CON (*p* < 0.01, d: −1.74 to −1.76; Figure 6I). Although similar tendencies were observed for the other parameters during the single phase (i.e., higher contraction time/peak torque ratio, and lower TT torques and RTD specifically during the first 40 contractions in CWI vs. CON conditions), comparisons between the two conditions did not reach the level of significance (Figures 6A, C, G). Similarly, tendencies to higher TT torques, RTD and RTR, and tendencies to lower contraction time/peak torque ratio and HRT were noticeable in IPP-CWI compared with SP-CWI during the first 40 contractions, but Sidak’s multiple comparisons tests did not reveal any significant differences (Supplementary Table S1). Torque fatigue index and changes in contractile properties during the fatiguing protocol were not different between SP-CON and IPP-CON (Table 2). In contrast, torque fatigue index, and the increases in contraction time/peak torque ratio and in HRT were lower in SP-CWI than in IPP-CWI (*p* < 0.02, d: −0.82 to −0.88), while they were in the same range after IPP-CWI and after CON conditions. The decreases in RTD and RTR during the fatiguing protocol were not significantly different between SP-CWI and IPP-CWI.

## 4 Discussion

To our knowledge, this is the first study investigating the impact of moderate muscle cooling induced by single and intermittent/prolonged CWI exposures on muscle force production and muscle contractility in unfatigued state and during repeated electrically induced contractions in humans. In the unfatigued state, both SP-CWI and IPP-CWI reduced P100 and TT torques, and increased P20/P100 ratio, but the changes from baseline were less pronounced in IPP-CWI than in SP-CWI. Overall, moderate muscle cooling slowed down muscle contraction and relaxation to a lesser extent after IPP-CWI than SP-CWI. Muscle cooling neither affected MVIC torque nor CAR, while the reductions of KE and KF isokinetic torques by cooling were less marked after IPP-CWI than SP-CWI. During the early phase of the fatiguing protocol, SP-CWI impaired muscle contractile properties (especially HRT and RTR) compared with SP-CON, while IPP-CWI did not affect these parameters. During the fatiguing protocol, torque fatigue index and the changes in muscle contractile properties were larger (with *p* < 0.05 only found for contraction time/peak torque ratio and HRT) after IPP-CWI than SP-CWI, but were in the same range as after CON conditions. These differences between the two cooling modalities were accompanied by a lower reduction of superficial Tmu and a smaller increase in systolic BP directly after intermittent/prolonged than single CWI, suggesting a reduced vasoconstriction response after intermittent/prolonged cold water immersions.

### 4.1 Impact of moderate muscle cooling on MVIC torque and CAR in unfatigued state

Our results showed that moderate muscle cooling, as evidenced by a ∼4°C decline in Tmu at 3 cm depth, did not impair MVIC. This result is in contrast with previous studies including voluntary isometric contractions of the KE muscles, in which moderate muscle cooling reduced MVIC torque by ∼6% (Bergh and Ekblom, 1979) and ∼20% (Brazaitis et al., 2011) compared with the control condition. In Brazaitis et al. study (Brazaitis et al., 2011), the torques developed by the male participants in the control condition were ∼20% higher than those reported in the current study, and some methodological aspects were different compared to this work (e.g., KE performed with a different knee joint angle, different dynamometers used, different CWI protocols, etc.). Although it remains unclear, these differences might explain the discrepancy between the two studies. Previous works indicated that MVIC force declined by 1%–2% for every 1°C of decreasing Tmu (Asmussen et al., 1976; Bergh and Ekblom, 1979), while others proposed that maximal isometric force production is relatively stable within the muscle temperature range from 27°C to 40°C (Clarke et al., 1958; Petrofsky and Phillips, 1986). Altogether, the moderate reduction of Tmu observed in the current study was not sufficient to impair MVIC torque production. Furthermore, and in line with previous studies (Brazaitis et al., 2014b; Spillane and Bampouras, 2021), we observed that CWI did not affect voluntary activation (assessed from CAR) during MVIC, providing additional evidence that muscle cooling does not impact motor drive when the muscle is unfatigued.

### 4.2 Impact of moderate muscle cooling on maximal isokinetic torque and muscle contractile properties in unfatigued state

In our study, moderate muscle cooling impaired maximal isokinetic torque, a result previously observed in the KE muscles (Bergh and Ekblom, 1979), and in other types of dynamic exercises (Asmussen et al., 1976). To our knowledge, this is the first time that it is reported during isokinetic contractions of the KF muscles. Interestingly, the torque decline was more pronounced for KF-isoK contraction than for KE-isoK contraction (−23% and 11% at the end of SP-CWI, respectively). This may result from the faster contraction profile of the hamstrings muscle compared to the quadriceps muscle (Garrett et al., 1984), which may make the hamstring muscle more sensitive to thermal changes (Bennett, 1984). Muscle cooling particularly impairs maximal concentric strength because peak torque is only reached at a specific joint angle during the movement, and thus the time available to attain peak torque is much shorter than during MVIC (Bergh and Ekblom, 1979). Muscle cooling may extend the time required to fully activate the motor units due to lower nerve conduction velocity (Algafly and George, 2007). In addition, muscle cooling could reduce the speed of chemical reactions, leading to a slower excitation of the sarcolemma (Brazaitis et al., 2016), a slower rate of Ca^2+^ release (and uptake) from the sarcoplasmic reticulum (Kössler et al., 1987), and a delay in the formation (and dissociation) of the cross-bridges (Faulkner et al., 1990). These changes would consequently reduce the rate of force development and ability to quickly reach peak torque during dynamic movements. Furthermore, some studies have demonstrated that severe muscle cooling impairs muscle contractile properties and induces a left shift of the stimulation frequency-isometric force relationship (Davies et al., 1982; de Ruiter et al., 1999). In the current study, we provided clear evidence that moderate muscle cooling also elicits a shift towards a slower muscle contractile profile, as shown by an increase in P20/P100 ratio and by altered muscle contractile properties (increased contraction time/peak torque ratio and HRT, decreased RTR). These changes are certainly the result of the peripheral muscle effects mentioned above and are most likely independent of changes in Trec (Giesbrecht et al., 1995). The fact that P20 torque was not affected by moderate cooling, while P100 torque was reduced, suggests that actomyosin sensitivity to Ca^2+^ was probably not impaired by cooling in our study (de Ruiter et al., 1999).

### 4.3 Comparison of the changes in muscle contractile function between SP-CWI and IPP-CWI in unfatigued state and during the development of fatigue

In accordance with our hypothesis, we showed that the changes in muscle contractile function observed in response to moderate muscle cooling were less pronounced after IPP-CWI than SP-CWI. This was observed in unfatigued state for KE- and KF-isoK torques, P100 and TT torques, P20/P100 ratio, and muscle contractile properties (HRT, RTD, and RTR), but not for MVIC torque. Compared with the CON condition, SP-CWI impaired muscle contractile properties (especially HRT and RTR) during the early phase of the fatiguing protocol, while IPP-CWI did not substantially affect these parameters. Upon cold exposure, early physiological responses include cutaneous vasoconstriction and reduced skin blood flow, leading to decreased convective heat transfer between the body’s core and shell (i.e., skin, subcutaneous adipose tissue, and skeletal muscle), which results in an effective maintenance of core temperature (Castellani and Young, 2016). In our study, the reduction of Tmu observed superficially (at 1 cm depth) was less pronounced after IPP-CWI than SP-CWI (−4.83°C vs. −9.73°C, respectively), and the changes in systolic BP were lower after IPP-CWI than SP-CWI. Although skin temperature and cardiac output were not assessed in this study, our findings suggest that peripheral resistance and cutaneous vasoconstriction response were likely lower after intermittent/prolonged compared with single cooling. These physiological adjustments may have led to an increased supply of warm blood from the core to the cutaneous and superficial muscle vessels, which consequently limited the reduction of Tmu (superficially) and decreased body insulation and core temperature during IPP-CWI (Tansey and Johnson, 2015). Several factors could explain this physiological response to intermittent cooling and referred as cold habituation (Castellani and Young, 2016). For instance, a reduced sensitivity of the thermoreceptors [transient receptor potential (TRP) channels], and a decreased discharge frequency of cold-sensitive thermoreceptors may result in a reduced neural flux to the neural system for thermoregulatory response (Hensel and Zotterman, 1951; Vybíral et al., 2000; Brazaitis et al., 2014a). Fatigue of smooth muscle has been previously reported (Yamanishi et al., 1994), suggesting that this could also occur in vascular smooth cells during intermittent/prolonged cooling, which may have compromised the vasoconstriction response (Castellani et al., 1998). Furthermore, it is unlikely that sitting at thermoneutral room temperature has evoked superficial vasodilatation because TRPM8 cold channels are normally activated at temperature below 27°C (Fujita et al., 2013). It is however possible that muscle contractions and dynamic movements from the bath to the chair (and *vice versa*) and on the dynamometer induced small changes in muscle blood perfusion, but they were insufficient to affect Tmu, as evidenced by the absence of differences in Tmu between 150 and 165 min (i.e., directly before and after the fourth neuromuscular testing) in CWI condition (see Figure 2J).

As shown in Table 2, torque fatigue index and the changes in contractile properties (contraction time/peak torque ratio and HRT) during repeated contractions induced by high frequency electrical stimulation were more elevated after intermittent/prolonged-CWI than after single-CWI but were in the same range as after CON conditions. This result indicates that a moderate and homogenous reduction of Tmu (4°C–5°C at the three depths) after intermittent/prolonged CWI has no major effect on muscle fatigability and contractility during repeated contractions, while these Tmu changes led to a mild impairment of muscle contractile function in unfatigued state. The homogenous (4°C–5°C) and heterogeneous (4°C–10 °C) decline in Tmu after intermittent/prolonged and single cooling, respectively, most likely explain the differences in functional outcomes observed in this study. Differences in muscle blood perfusion between the two cooling modalities, especially at the more superficial layers, might be another explanatory factor. Indeed, recent findings indicate that changes in muscle perfusion in response to CWI at low temperature (8 °C for 10 min), although modest, are not uniform across the quadriceps muscle (Mawhinney et al., 2020).

### 4.4 Limitations

One aspect that remains unclear is whether cold habituation (i.e., lower decline in peripheral Tmu and potential reduction of vasoconstriction response) observed in this study is the result of intermittent cooling, prolonged body cold exposure, or the combination of both. As explained in the method section, intermittent CWI was used to induce a moderate and relatively stable reduction of Tmu in the deep portion of the vastus lateralis muscle, while avoiding hypothermia and too much discomfort for the participants. Cold habituation has been observed during repeated CWI (3 times 2 h with 2 h break; whole-body CWI at 20°C) where Trec was fully recovered before each cold bath (Castellani et al., 1998). Although the exact cause of cold habituation remains unclear, our study suggests that cold habituation could also occur when core temperature is reduced. As shown in this study, the reduction of superficial Tmu was larger than that of deep Tmu after SP-CWI, while the reduction of Tmu was homogeneous across the 3 depths after IPP-CWI. To be able to clearly answer whether the duration of muscle cooling affects muscle contractile function, an adequate protocol in which the reduction of both superficial and deep Tmu remain stable over several hours would be required. In addition, additional physiological parameters, including skin temperature, cardiac output, and cutaneous and muscle blood flow could be examined to further investigate vasoconstriction response. Furthermore, although Trec is almost exclusively used in the literature to estimate core temperature in response to CWI, it may underestimate the cooling rates (Miller et al., 2017). Finally, core temperature (estimated from Trec) seems to decrease faster in females than males in response to CWI following hyperthermia, which may be due to several factors, including sex-differences in body surface area, lean body mass, and body surface area to lean body mass ratios (Boehm and Miller, 2019). Therefore, it is possible that changes in Tmu in response to cooling are different between females and males, which may influence the outcomes of muscle contractile function assessed in this study.

### 4.5 Conclusion

We provide evidence that moderate muscle cooling impairs force production during electrically stimulated contractions at high frequency and during voluntary isokinetic contractions, but not during MVIC. In addition, it slows down muscle contractile properties in unfatigued state and during the early phase of the fatiguing protocol. Our results indicate that in comparison with single CWI, intermittent/prolonged CWI has a less severe impact on muscle function in unfatigued state. In contrast, during repeated contractions, muscle fatigability and the changes in muscle contractile properties are larger after IPP-CWI than after SP-CWI but are in the same range as after CON conditions. We believe that intermittent/prolonged CWI induces a less pronounced fast-to-slow contractile transition compared to single CWI, and this physiological response may result from the reduced vasoconstriction response and enhanced blood perfusion of the superficial layers of the muscle, which could ultimately limit the reduction of superficial Tmu.

## Data Availability

The original contributions presented in the study are included in the article/Supplementary Material, further inquiries can be directed to the corresponding authors.
