# Supplementary material for: Moderate muscle cooling induced by single and intermittent/prolonged cold-water immersions differently affects muscle contractile function in young males
Source: Front Physiol. 2023 Mar 21;14:1172817. doi: 10.3389/fphys.2023.1172817 (PMC10070757; doi:10.3389/fphys.2023.1172817)
Supplement: Supplementary file 1 [file Table1.docx]

**Supplementary Table S1. Changes in torques and contractile properties during the fatiguing protocol**

|  |  | Contraction number | | | | | |  |  |  |
| --- | --- | --- | --- | --- | --- | --- | --- | --- | --- | --- |
|  |  | 1 to 3 | 4 to 20 | 21 to 40 | 41 to 60 | 61 to 80 | 81 to 100 | Contraction number effect | Phase effect | Interaction |
| TT Torque (Nm) | SP-CON | 149.2 ± 54.5 | 135.7 ± 46.8 | 118.0 ± 34.9 | 93.8 ± 22.4 | 74.4 ± 15.0 | 64.8 ± 13.8 | < 0.001 | 0.92 | 0.55 |
|  | IPP-CON | 153.6 ± 66.4 | 138.1 ± 60.5 | 118.8 ± 41.6 | 92.9 ± 20.6 | 72.2 ± 13.6 | 63.2 ± 12.4 |  |  |  |
|  | SP-CWI | 143.3 ± 55.3 | 129.3 ± 47.3 | 112.4 ± 37.4 | 91.9 ± 24.6 | 76.8 ± 15.9 | 68.8 ± 14.0 | < 0.001 | 0.18 | 0.02 |
|  | IPP-CWI | 155.9 ± 46.2 | 139.9 ± 41.7 | 122.2 ± 34.0 | 98.4 ± 22.9 | 78.7 ± 15.7 | 68.4 ± 15.0 |  |  |  |
| Contraction time/peak torque (ms/Nm) | SP-CON | 2.06 ± 0.70 | 2.15 ± 0.66 | 2.46 ± 0.74 | 3.10 ± 1.05 | 3.74 ± 1.01 | 4.19 ± 0.97 | < 0.001 | 0.94 | 0.64 |
|  | IPP-CON | 2.07 ± 0.66 | 2.20 ± 0.72 | 2.49 ± 0.70 | 2.99 ± 0.77 | 3.72 ± 0.89 | 4.20 ± 1.01 |  |  |  |
|  | SP-CWI | 2.24 ± 0.69 | 2.45 ± 0.73 | 2.75 ± 0.74 | 3.17 ± 0.73 | 3.60 ± 0.71 | 3.96 ± 0.77 | < 0.001 | 0.23 | 0.02 |
|  | IPP-CWI | 1.98 ± 0.51 | 2.20 ± 0.59 | 2.47 ± 0.57 | 2.91 ± 0.49 | 3.50 ± 0.55 | 4.02 ± 0.78 |  |  |  |
| HRT time (ms) | SP-CON | 85.6 ± 21.1 | 99.4 ± 20.4 | 120.8 ± 21.9 | 150.6 ± 30.4 | 173.7 ± 42.0 | 181.4 ± 47.5 | < 0.001 | 0.94 | 0.49 |
|  | IPP-CON | 82.2 ± 12.7 | 100.2 ± 22.7 | 124.9 ± 25.0 | 156.1 ± 33.0 | 172.4 ± 31.5 | 177.9 ± 35.8 |  |  |  |
|  | SP-CWI | 108.9 ± 20.1 | 112.9 ± 15.5 | 132.3 ± 13.0 | 157.6 ± 21.3 | 170.4 ± 34.0 | 174.3 ± 45.3 | < 0.001 | 0.77 | 0.008 |
|  | IPP-CWI | 95.6 ± 21.7 | 99.5 ± 13.4 | 123.5 ± 17.3 | 158.0 ± 30.5 | 181.0 ± 46.2 | 188.3 ± 52.2 |  |  |  |
| RTD (Nm/s) | SP-CON | 1946.7 ± 536.5 | 1865.9 ± 499.9 | 1571.2 ± 364.0 | 1135.8 ± 235.9 | 860.1 ± 211.7 | 749.9 ± 206,7 | < 0.001 | 0.09 | 0.78 |
|  | IPP-CON | 2145.3 ± 708.7 | 2076.1 ± 664.9 | 1772.9 ± 453.8 | 1274.8 ± 301.0 | 1033.8 ± 357.3 | 887.4 ± 333.1 |  |  |  |
|  | SP-CWI | 1654.3 ± 417.2 | 1518.9 ± 401.9 | 1292.6 ± 310.9 | 1013.7 ± 211.0 | 827.2 ± 180.9 | 747.2 ± 176.0 | < 0.001 | 0.06 | 0.008 |
|  | IPP-CWI | 1880.7 ± 493.2 | 1874.2 ± 553.2 | 1595.3 ± 438.9 | 1161.2 ± 288.3 | 890.0 ± 239.2 | 763.7 ± 221.6 |  |  |  |
| RTR (Nm/s) | SP-CON | 1547.7 ± 581.6 | 1397.0 ± 518.8 | 938.3 ± 257.5 | 587.5 ± 200.0 | 433.9 ± 177.3 | 373.3 ± 153.3 | < 0.001 | 0.12 | 0.20 |
|  | IPP-CON | 1394.3 ± 436.4 | 1251.8 ± 357.9 | 861.8 ± 228.3 | 581.8 ± 161.7 | 401.1 ± 131.6 | 332.2 ± 104.4 |  |  |  |
|  | SP-CWI | 976.3 ± 359.7 | 964.8 ± 363.3 | 692.4 ± 207.2 | 478.6 ± 115.8 | 380.7 ± 99.4 | 364.5 ± 103.4 | < 0.001 | 0.03 | 0.007 |
|  | IPP-CWI | 1317.7 ± 501.6 | 1176.8 ± 413.8 | 851.1 ± 289.7 | 575.7 ± 185.8 | 450.6 ± 195.9 | 412.7 ± 208.9 |  |  |  |

TT, 250-ms test train stimulation at 100 Hz; HRT, half-relaxation time; RTD, peak rate of torque development; RTR, peak rate of torque relaxation; SP, single phase; IPP, intermittent/prolonged phase.

Contractile properties (i.e., contraction time/peak torque, HRT, RTD and RTR) are derived from the TT stimulations. The table presents the average values obtained between the 1^st^ and 3^rd^ contractions, the 4^th^ and 20^th^ contractions, the 21^st^ and 40^th^ contractions, the 41^st^ and 60^th^ contractions, the 61^st^ and 80^th^ contractions, and the 81^st^ and 100^th^ contractions. Data are shown as mean ± SD.

N=12 for all parameters except for RTD and RTR (N=10)
