# Supplementary figures and images for: Moderate muscle cooling induced by single and intermittent/prolonged cold-water immersions differently affects muscle contractile function in young males
Source: Front Physiol. 2023 Mar 21;14:1172817. doi: 10.3389/fphys.2023.1172817 (PMC10070757; doi:10.3389/fphys.2023.1172817)

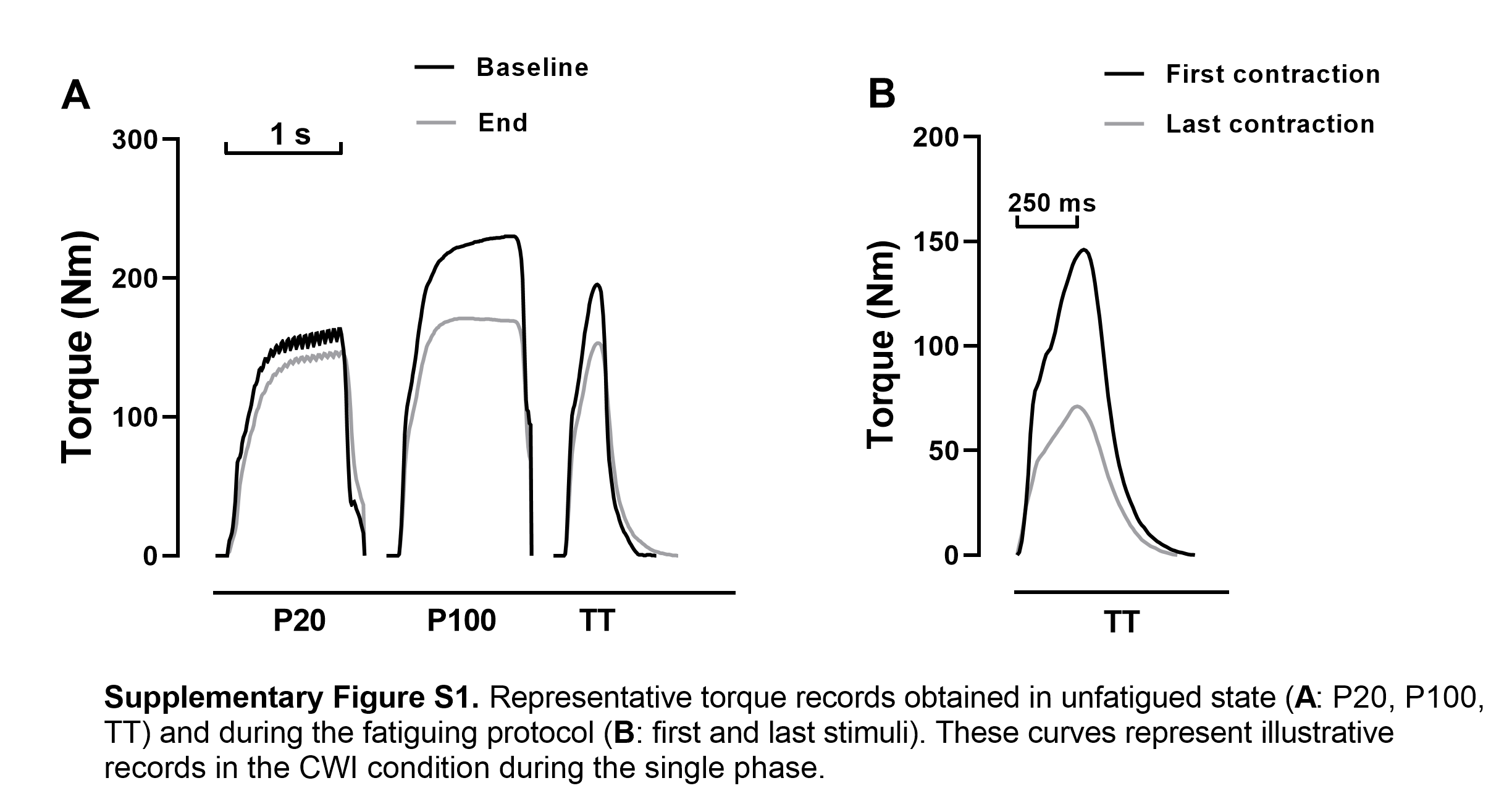

Supplement: Supplementary file 2 [file Image1.tif]
